# Engineering and Technological Advancements in Repetitive Transcranial Magnetic Stimulation (rTMS): A Five-Year Review

**DOI:** 10.3390/brainsci14111092

**Published:** 2024-10-30

**Authors:** Abigail Tubbs, Enrique Alvarez Vazquez

**Affiliations:** Biomedical Engineering, College of Engineering and Mines, University of North Dakota, Grand Forks, ND 58202, USA; enrique.vazquez@und.edu

**Keywords:** repetitive transcranial magnetic stimulation (rTMS), neuromodulation, brain–computer interface (BCI), magnetic coil technology, personalized medicine, artificial intelligence (AI), neuroimaging, psychiatric treatment, device design, treatment protocols, safety protocols, technological advancements, portable devices, clinical efficacy

## Abstract

In the past five years, repetitive transcranial magnetic stimulation (rTMS) has evolved significantly, driven by advancements in device design, treatment protocols, software integration, and brain-computer interfaces (BCIs). This review evaluates how these innovations enhance the safety, efficacy, and accessibility of rTMS while identifying key challenges such as protocol standardization and ethical considerations. A structured review of peer-reviewed studies from 2019 to 2024 focused on technological and clinical advancements in rTMS, including AI-driven personalized treatments, portable devices, and integrated BCIs. AI algorithms have optimized patient-specific protocols, while portable devices have expanded access. Enhanced coil designs and BCI integration offer more precise and adaptive neuromodulation. However, challenges remain in standardizing protocols, addressing device complexity, and ensuring equitable access. While recent innovations improve rTMS’s clinical utility, gaps in long-term efficacy and ethical concerns persist. Future research must prioritize standardization, accessibility, and robust ethical frameworks to ensure rTMS’s sustainable impact.

## 1. Introduction

Repetitive transcranial magnetic stimulation (rTMS) is a non-invasive brain stimulation technique that uses magnetic fields to modulate neural activity in targeted brain regions [1]. Since its inception in the 1980s, rTMS has become a significant tool in clinical and research settings, particularly neurology and psychiatry. Its ability to influence brain activity without direct physical intervention has made it a valuable alternative to more invasive treatment methods. The rTMS technique involves placing an electromagnetic coil near the scalp, generating brief magnetic pulses, and indirectly stimulating the brain neurons [2]. The foundational principles of rTMS trace back to Michael Faraday’s discovery of electromagnetic induction in 1831, which laid the groundwork for modern magnetic stimulation technologies [3]. However, applying this principle to brain stimulation only emerged in the late 20th century when researchers began exploring the therapeutic potential of magnetic fields in modulating neural activity [4]. Early experiments with rTMS were primarily focused on neurophysiological studies, providing insights into the functioning of the motor cortex and other brain regions [5]. These studies laid the groundwork for understanding how magnetic pulses can influence neuronal activity and paved the way for clinical applications. The increasing use of rTMS in clinical settings has been driven by its ability to target specific brain regions precisely, minimizing the side effects commonly associated with pharmacological treatments [6]. Recent advancements in coil design and stimulation protocols have further enhanced the precision and efficacy of rTMS, making it an increasingly attractive option for clinicians and researchers alike [7]. The technique’s non-invasive nature, combined with the growing body of evidence supporting its efficacy, has positioned rTMS as a cornerstone in treating neuropsychiatric disorders, with ongoing research aimed at expanding its applications and improving its outcomes [8].

In the last five years, several rapid developments have unfolded, driven by clinical demand and innovation [9]. Despite the extensive literature on this technology, there remains a critical need to understand how recent technological innovations collectively impact clinical practice [10]. Previous reviews have often focused solely on specific aspects of rTMS, such as clinical applications, device components, or theoretical mechanisms, leading to an isolated comprehension of single aspects of the technology [11]. In contrast, this review takes a multi-faceted approach, examining various advancements, including coil design innovations, AI-driven treatment protocols, and developing portable devices within a unified framework. This work seeks to offer a more cohesive understanding of how these innovations are interrelated and how they collectively enhance the efficacy, safety, and accessibility of rTMS.

In addition to technological advancements, this review addresses the ethical considerations and practical challenges associated with implementing these innovations. This paper aims to fill a significant gap in the existing literature by offering a holistic analysis of the latest developments. It provides a forward-looking perspective essential for researchers, clinicians, and engineers aiming to drive the next wave of innovations in neuromodulation therapies [12]. Furthermore, the review examines recent connectivity and user interface innovations, which have enabled more interactive and personalized patient treatment sessions. The emergence of AI-driven algorithms for optimizing treatment protocols represents a leap forward in our ability to specify the therapeutic outcomes of rTMS [13]. Additionally, the trend toward miniaturization has led to the development of more compact and portable rTMS devices, potentially expanding access to this treatment in non-traditional settings and among broader patient demographics [14]. These technological improvements have opened new avenues for research and application, promising to reshape the future of psychiatric and neurological therapy with enhanced efficacy [15,16].

## 2. Methodology

This review employed a structured approach to identify and analyze recent advancements in repetitive transcranial magnetic stimulation (rTMS), focusing on both technological developments and clinical applications. The search was conducted using Google Scholar, with the filter set to include studies published between 2019 and 2024. This 5-year timeframe was chosen to capture the most recent trends and innovations relevant to rTMS. Several targeted search terms were used to identify studies across technological and clinical domains, including “recent rTMS advancements”, “coil advancements in rTMS”, “clinical applications of rTMS”, “technological innovations in rTMS”, and “recent trends in rTMS”. The focus was on identifying peer-reviewed articles to guarantee the reliability of the findings. Studies from diverse disciplines, including neurology, psychiatry, and biomedical engineering, were considered to provide an overview of rTMS’s multidisciplinary impact. Clinical trials and case studies were chosen based on their relevance to ongoing rTMS research and the availability of recent clinical data. Depression, bipolar disorder, and behavioral addictions were selected to provide a comprehensive view of rTMS’s efficacy across both established and emerging therapeutic domains. Depression was chosen due to its extensive history of related studies and well-documented use of rTMS as a treatment. Bipolar disorder and behavioral addictions were included as they represent emerging areas of research where rTMS has shown variable outcomes, offering insight into both its potential and limitations [17]. This selection provided a balanced understanding of rTMS applications in psychiatric care.

The selection process followed a multi-step protocol. Titles and abstracts were initially reviewed to determine whether the study aligned with the technological and clinical focus of the review. The discussion sections were examined for articles that passed the initial screening to assess the study’s key findings and contributions. A full-text analysis was then performed on the selected articles to extract detailed insights into coil technology, software integration, treatment protocols, and clinical outcomes. Studies were included if they reported technological advancements in rTMS devices, coil design, or software systems, focused on clinical applications such as patient outcomes and treatment protocols, or discussed emerging trends and innovations shaping the future of rTMS technology. Articles were excluded if they were not peer-reviewed, lacked technical rigor, did not focus on rTMS, or were unavailable in English. Relevant information from the selected articles was categorized based on technological advancements, treatment protocols, clinical outcomes, and safety considerations. Key trends and challenges were identified, along with opportunities for future research.

## 3. Background and Current State of rTMS Technology

The development of repetitive transcranial magnetic stimulation (rTMS) is rooted in the fundamental principles of electromagnetism, first discovered by Michael Faraday in 1831 [18]. Faraday’s Law, which describes how a magnetic field is generated around a coil when an electric current passes through it, laid the groundwork for various electromagnetic technologies, including those used in medical science [19]. However, it was not until 1985 that Anthony Barker and his colleagues at the University of Sheffield successfully demonstrated the application of transcranial magnetic stimulation (TMS) in humans [20]. This breakthrough involved non-invasive motor cortex stimulation, leading to observable muscle contractions, and proved that magnetic fields could safely and effectively influence neural activity in the brain [21]. This discovery opened new avenues for neuroscientific research and clinical applications [22].

Initially, TMS was used primarily as a diagnostic tool to study nerve conduction and brain function. However, as researchers recognized its potential to modulate brain activity, the focus gradually shifted toward therapeutic applications [23].

**Figure 1:**
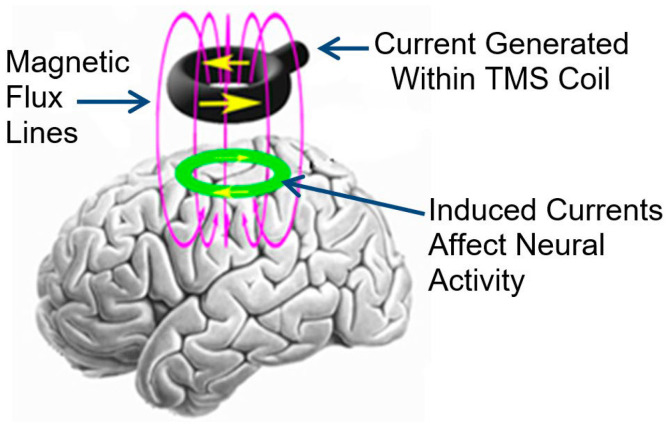
Illustration of how transcranial magnetic stimulation (TMS) induces electric currents in the brain. The TMS coil generates a magnetic field (depicted by the magnetic flux lines), which induces electrical currents within the brain tissue, affecting neural activity. This process forms the basis for both diagnostic TMS and therapeutic repetitive TMS (rTMS), the latter used for various neurological and psychiatric conditions [24].

**Figure 2:**
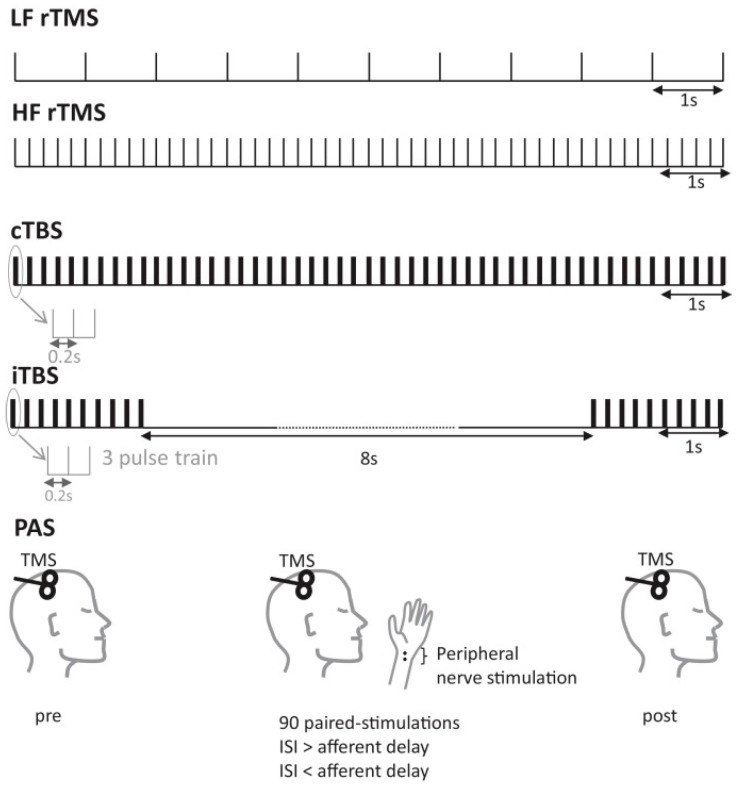
Comparison of different repetitive transcranial magnetic stimulation (rTMS) protocols, including continuous theta-burst stimulation (cTBS), intermittent theta-burst stimulation (iTBS), low-frequency rTMS (LF rTMS), and high-frequency rTMS (HF rTMS). The diagram also shows the basic setup of an rTMS session, highlighting the placement of the TMS coil over the head and the potential for paired peripheral nerve stimulation (PAS) to enhance therapeutic outcomes. In PAS, the interstimulus interval (ISI) represents the time between the TMS pulse and the peripheral nerve stimulation to optimize the therapeutic outcomes [26].

Unlike diagnostic TMS, which requires a second coil, rTMS simplifies the process by transferring the magnetic field from a single coil to the brain, stimulating nerve cells directly [24]. The process is visually represented in Figure 1 [25], where the magnetic flux lines generated within the TMS coil induce currents in the brain, affecting neural activity. The magnetic fields generated by rTMS create secondary electric currents in the brain, altering the electrical activity of neurons [26]. As shown in Figure 2, different protocols of rTMS (such as continuous theta-burst stimulation [cTBS], intermittent theta-burst stimulation [iTBS], low-frequency rTMS [LF rTMS], and high-frequency rTMS [HF rTMS]) are utilized depending on the therapeutic needs. These protocols can induce magnetic fields up to two centimeters deep, reaching regions between the white and gray matter and causing neurophysiological changes that have therapeutic effects [27]. The concept of using repetitive pulses, now known as rTMS, emerged as researchers observed that the impact of TMS could be prolonged with repeated stimulation [28]. By the late 1900s and early 2000s, rTMS began to be studied extensively as a treatment for depression, where it showed potential as an alternative to traditional treatments like medication and electroconvulsive therapy [29]. The FDA’s clearance of rTMS in 2008 for treatment-resistant depression marked a significant milestone, validating its efficacy and safety [30]. Since then, the scope of rTMS applications has continued to expand. It is now being investigated and used in the treatment of a variety of conditions, including stroke rehabilitation, Parkinson’s disease, schizophrenia, and obsessive-compulsive disorder [31]. Each application leverages the ability of rTMS to either stimulate or suppress neural activity in targeted brain regions, depending on the frequency of the pulses used [32].

The basic principle of rTMS involves generating a magnetic field by passing a current through a coil near the scalp [33]. This magnetic field induces an electric current in the underlying neural tissue, leading to depolarization or hyperpolarization of neurons, depending on the stimulation parameters [34]. The current passing through the TMS coil typically ranges from 5000 to 10,000 amperes (A), which is necessary to generate a sufficiently strong magnetic field. The inductance of a typical TMS coil is usually within the range of 10 to 20 microhenries (µH) [35]. The equipment used for rTMS typically includes a transformer that charges a capacitor, which then discharges to create a magnetic pulse in the coil [26]. A sub-circuit controls the pulses’ temperature, ensuring effective stimulation. A high-voltage electrical switch creates a short pulse (about 250 microseconds) for effective stimulation [26]. This setup allows the system to produce magnetic fields with a strength of up to 1.5 to 2.5 teslas (T), sufficient to modulate neural activity effectively [36].

rTMS has been widely used in clinical and research settings for various purposes, including the treatment of depression, the facilitation of stroke recovery, and the study of brain function and neuroplasticity [37]. Recent advancements in rTMS technology have focused on improving coil design, refining stimulation protocols, and integrating rTMS with neuroimaging techniques such as functional magnetic resonance imaging (fMRI) and electroencephalography (EEG) [38]. These advancements have significantly enhanced the precision, efficacy, and comfort of rTMS treatments, broadening their applicability and accessibility in clinical practice. The historical development of rTMS underscores the transformative impact of electromagnetic principles on medical science, particularly in neuromodulation [39].

Standard protocols typically vary in frequency, with high-frequency rTMS (>1 Hz) generally used to increase cortical excitability and low-frequency rTMS (≤1 Hz) used to decrease it [40]. The choice of frequency and other parameters like pulse duration and intensity are critical in shaping the therapeutic outcomes of rTMS [41]. Clinicians can tailor treatments to meet the specific needs of patients by adjusting these parameters, allowing for precise targeting of brain regions and functions. However, the effectiveness of rTMS can be limited by individual variations in brain anatomy and physiology, which may affect the accuracy of target region stimulation and the consistency of treatment outcomes [42]. Before the last five years, significant developments in rTMS technology included advancements in coil design, leading to more focused and deeper stimulation and integrating rTMS with neuroimaging techniques [43]. These innovations have expanded the therapeutic potential of rTMS, improved our understanding of its mechanisms of action, and paved the way for further research and innovation in neuromodulation therapies [44].

## 4. Recent Technological Advancements in rTMS Devices

### 4.1. Innovations in Device Design and Functionality

In the last five years, significant innovations have been made in the design and functionality of rTMS devices [45]. These advances include improvements in the power circuits of TMS units, enabling more control over pulse characteristics [46]. The introduction of insulate gate bipolar transistors (IGBTs) and metal–oxide–semiconductor field-effect transistors (MOSFETs) in newer rTMS devices has enhanced the flexibility and precision of stimulation, allowing for a broader range of frequencies and pulse shapes [47]. However, these advancements come with increased complexity and cost, heat generation, higher maintenance requirements, optimization difficulties, and potential safety concerns.

These innovations have introduced several challenges, despite their technological progress. The increased complexity of devices may lead to higher costs and maintenance burdens, limiting their accessibility in resource-constrained settings [48]. Additionally, their generation of excess heat and the need for precise optimization can complicate device operation and longevity. Safety concerns also arise when more powerful circuits are implemented, necessitating robust safety protocols to prevent adverse effects [48].

To address these limitations, ongoing research should focus on developing more efficient cooling systems to manage heat generation and exploring cost-effective materials that maintain device performance while reducing costs [49]. Furthermore, AI and machine learning advancements could be leveraged to optimize device settings automatically, reducing the burden on clinicians and ensuring safer operation [50]. Future research should also explore modular device designs that allow for easier maintenance and upgrades, potentially extending the lifespan and usability of rTMS devices in various clinical settings. In clinical settings, there is a growing demand for patient-friendly usage, which necessitates devices that are more user-friendly, comfortable, and accessible [51]. This includes the development of more compact and portable devices and user interfaces that are intuitive and easy to navigate for clinicians and patients. However, achieving this balance between innovation and practicality remains a significant challenge [49].

An open-access database has been created to compare commercial and non-commercial TMS devices, helping researchers and clinicians select suitable devices and protocols for their specific needs [50]. For researchers, the database facilitates the identification of devices that offer the necessary precision and flexibility for experimental studies. It allows them to compare different models based on their capability to deliver specific pulse shapes, frequencies, and intensities, which are critical for investigating the effects of TMS on neural activity and behavior [52].

While this open-access database is a valuable resource, it has limitations. The database may need to be more comprehensive, potentially excluding newer or less standard devices [51]. The information provided might need to be updated or completed, affecting the accuracy of comparisons. Additionally, the database relies on user contributions and manufacturer specifications, which could introduce biases or inaccuracies. Access to detailed technical specifications and proprietary information may limit the comparison depth [53].

Future enhancements to the database could include integrating real-time data updates from manufacturers and including more standardized, peer-reviewed technical specifications. Expanding the database to include user reviews and clinical outcomes data could provide a more comprehensive tool for researchers and clinicians [50].

Table 1 highlights the key innovations in rTMS device design and functionality, their associated limitations, and reference numbers for further details.

### 4.2. Improvements in Magnetic Coil Technology

In 2019, two innovative TMS coils were introduced: the triple halo coil (TH Coil) for deep brain stimulation, and the quadruple butterfly coil (QBC) for precise spatial stimulation. The TH coil’s unique geometry allows for enhanced penetration and a magnetic field up to seven times stronger than conventional coils. It is ideal for treating deep brain conditions such as Parkinson’s and PTSD [54]. Conversely, the QBC focuses on precision, reducing the stimulation volume by about 10% compared to the figure-8 coil and incorporating passive magnetic shields to refine its focality further, making it particularly suitable for conditions requiring targeted stimulation, like schizophrenia [54]. Despite these advancements, both designs still face complexity and the need for further optimization, impacting their accessibility and clinical versatility [54].

The complexity of these new coil designs could hinder their widespread adoption. The TH coil, while powerful, may be too specialized for general use, limiting its applicability [54]. The QBC, though more precise, requires further optimization to improve its ease of use and integration into existing clinical workflows [54].

Addressing these challenges will require further research into simplifying coil designs without sacrificing performance. Innovations in material science could lead to more flexible and adaptable coil geometries. Additionally, developing more user-friendly software interfaces to accompany these advanced coils could enhance their clinical usability and reduce the learning curve for practitioners [54].

In 2020, a study confirmed the safety and efficiency of novel H-DC coils for transcranial magnetic stimulation, but they were not significantly superior to traditional figure-8 coils [55]. Additionally, another 2020 study introduced a novel air-core TMS coil, which demonstrated improved focality and enhanced electric field control, particularly in deep brain regions. This air-core coil design helps to reduce eddy currents and improve stimulation precision, making it a valuable addition to the repertoire of TMS coil technologies [56].

The H-DC coils, despite their innovative nature, did not show a significant advantage over existing technologies, suggesting that future research should focus on refining their design or identifying specific clinical scenarios where they might offer unique benefits [55]. While promising regarding focality and control, the air-core coil may require further development to ensure its practicality and effectiveness in diverse clinical settings [56].

A study in 2022 aimed to design an rTMS circular coil, comparing simulation results of magnetic field intensity (H) and flux density (B) at 0.5 and 1 Hz with theoretical calculations using CST Studio Suite [57]. The circular coil showed acceptable consistency between simulated and theoretical values for both H and B fields, suggesting its effectiveness for rTMS applications. However, limitations include the need for comparative data with similar coils on human head voxels, which could enhance the reliability of findings. Further research could involve simulations with different coil types, like figure-8 coils [57].

Comparative studies involving human head models are essential to validate these new coil designs fully [56]. Expanding simulation studies to include a broader range of coil types and brain regions could provide a more comprehensive understanding of their potential benefits and limitations [58].

**Figure 3:**
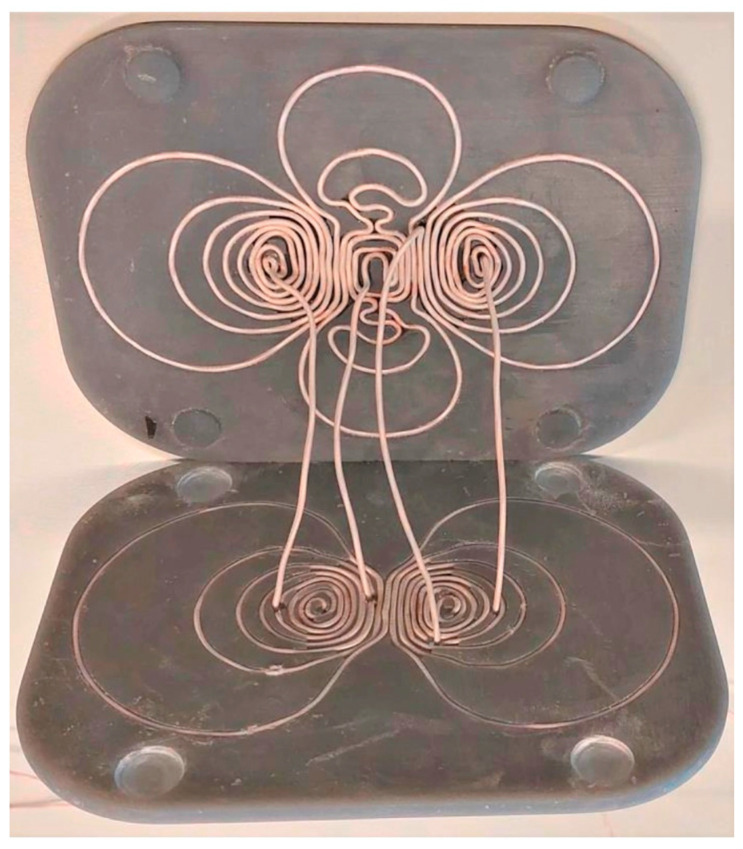
3D-printed multi-locus TMS coils [58].

**Figure 4:**
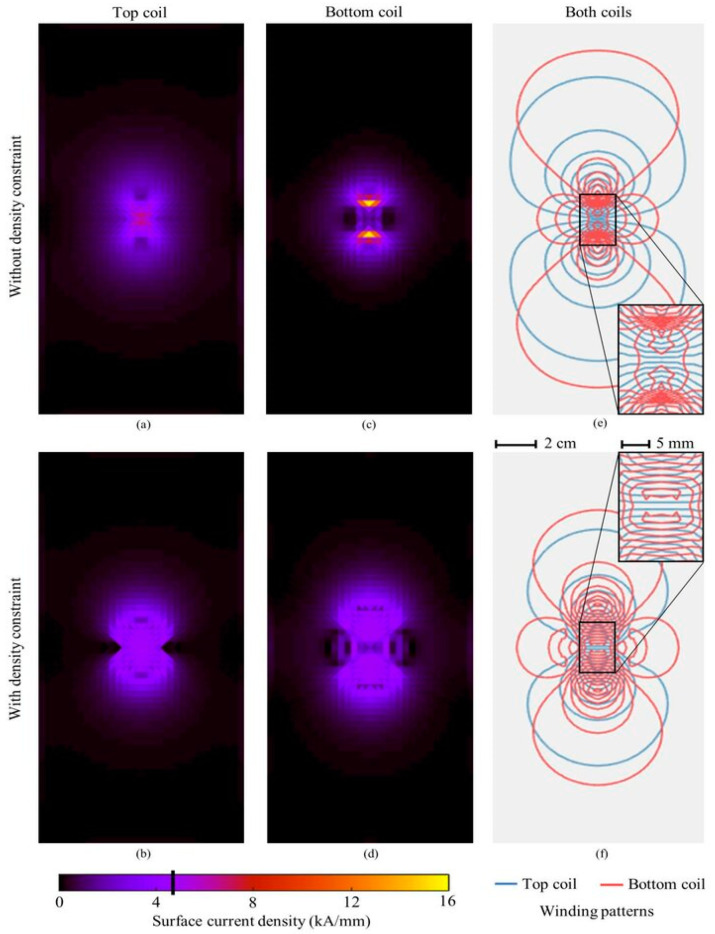
Illustrates the optimized surface current density patterns for the top and bottom coils used in rTMS, comparing configurations with and without density constraints. Subfigures (**a**,**c**) show the current distributions for the top and bottom coils without constraints, resulting in broader, less focused magnetic fields. In contrast, subfigures (**b**,**d**) depict the same coils with density constraints, demonstrating more compact and precise current patterns that enhance the focality and effectiveness of brain stimulation. Subfigure (**e**) overlays the magnetic field patterns from both coils—red for the top coil and blue for the bottom coil—highlighting how these optimized fields align for targeted stimulation. Finally, subfigure (**f**) zooms in on the winding patterns of both coils, emphasizing the intricate design required, with 2 cm and 5 mm spacing between windings, to generate accurate magnetic fields. These advancements in coil design improve the safety, precision, and efficacy of rTMS treatments by ensuring more controlled and localized brain stimulation [58].

In 2023, a study introduced a new design method for multi-locus transcranial magnetic stimulation coils, focusing on manufacturability and precise control over the electric field distributions [59]. This method significantly reduces optimization time and incorporates constraints for custom current density and electric field fidelity, ensuring target fields are accurately reproduced within feasible winding densities. Figure 3 showcases the two 3D-printed coils, where a single layer of wire was carefully wound into the grooves. The complex design of the bottom coil necessitated a detour through the top coil, with winding paths determined by isolines and manually inserted connecting paths to ensure proper current flow direction, as dictated by the surface current pattern [59]. As illustrated in Figure 4, the surface current density patterns for both the top and bottom coils, with and without density constraints, demonstrate the impact of advanced coil design on magnetic field distribution. Such optimizations allow for more targeted stimulation, reducing undesired stimulation of surrounding brain regions. The winding patterns (rightmost panel) further highlight the intricacies of modern coil geometry, with tighter winding contributing to enhanced focality and depth penetration of the magnetic field [59]. The limitations of the new coil design included potential issues with manufacturability due to high winding density and complex winding patterns, which can be challenging to replicate accurately in physical coils. Additionally, the method assumes a specific distance between wires to ensure feasibility, which may only sometimes be precisely achieved in practice, potentially affecting the coil’s performance [59]. Table 2 summarizes the benefits and limitations of recent advances in the coil technology discussed.

To overcome these limitations, future research could explore alternative manufacturing techniques, such as 3D printing, which might allow for more precise and reproducible coil designs. Developing more robust simulation tools that account for manufacturing variances could improve the reliability and performance of these advanced coils [55].

## 5. Advancements in Treatment Protocols

Significant advancements in rTMS treatment protocols and methodologies have been achieved in the past five years, indicating a pivotal shift towards more personalized and effective therapeutic approaches [60]. Developments have mainly focused on customizing treatment for individual patients, acknowledging the variability in brain anatomy and functional connectivity across individuals [61]. This customization has been facilitated by leveraging data from neuroimaging technologies such as fMRI and diffusion tensor imaging (DTI). This allows clinicians to target specific brain regions more accurately and adjust stimulation parameters to the patient’s unique neurophysiological profile [62].

Additionally, there has been a notable trend towards integrating rTMS with other therapies and diagnostic tools, creating a more holistic treatment approach. For instance, the combination of rTMS with cognitive behavioral therapy (CBT) has shown enhanced efficacy in treating depression, suggesting a synergistic effect between neuromodulation and psychotherapeutic interventions [63]. Using machine learning algorithms to analyze patient data has also enabled the prediction of treatment outcomes, further refining treatment protocols to maximize therapeutic success [64]. These advancements underscore a significant move towards more tailored, effective, and integrative treatment.

In 2022, fNIRS was used to measure the hemodynamic responses in the prefrontal cortex of healthy adult participants during a cognitive task, both before and after rTMS treatment [65]. The study aimed to investigate the effects of rTMS on cognitive performance and its potential as a therapeutic tool. fNIRS was chosen for its ability to non-invasively monitor changes in oxygenated and deoxygenated hemoglobin levels, which are indicators of neural activity. The results showed significant increases in oxygenated hemoglobin concentration after rTMS, suggesting enhanced neural activity and cognitive performance [65]. However, the study had limitations, including a small sample size and the lack of a sham rTMS control group, which could affect the generalizability and interpretation of the findings. Additionally, the study focused only on the prefrontal cortex, limiting the understanding of rTMS effects on other brain regions involved in cognition [65].

## 6. Software and Control Systems

### 6.1. Neuronavigational Systems and Advanced Software

Recent developments in rTMS technology have markedly improved the software used to control these devices. Modern rTMS systems are now equipped with sophisticated software allowing precise control over stimulation parameters such as the magnetic pulses’ intensity, frequency, and duration [66]. This has enabled clinicians to tailor rTMS treatments to patients’ needs, improving treatment outcomes. Integrating neuronavigational systems, which utilize MRI data to guide the positioning of the TMS coil, has further enhanced the precision and effectiveness of rTMS therapy [67]. These neuronavigational systems ensure that the magnetic pulses are accurately targeted at specific brain regions, which is crucial for treating various conditions [66]. However, the increased software sophistication can make the systems more complex, requiring specialized training and potentially limiting accessibility. While advantageous, the flexibility in setting stimulation parameters can challenge the standardization of treatment protocols. The effectiveness of treatments is heavily dependent on the reliability and accuracy of the software, with any bugs or errors potentially affecting safety and efficacy [66].

Additionally, the development and integration of advanced software can increase the overall cost of the devices, limiting accessibility in resource-limited settings [68]. Ensuring compatibility with existing hardware and integration with other technologies also presents challenges [69]. Addressing these limitations is crucial for the continued development and widespread adoption of advanced rTMS technology [70]. The user interface of rTMS devices has also seen significant improvements, making these systems more user-friendly and accessible to clinicians. The introduction of intuitive touch-screen interfaces and user-friendly software has streamlined the setup and administration of rTMS sessions, reducing the likelihood of operator error [71]. These advancements may only partially eliminate the potential for operator error, especially in complex clinical scenarios or with inexperienced users. Additionally, the increased use of digital interfaces raises concerns about cybersecurity and the protection of patient data, requiring robust security measures to safeguard sensitive information [71].

Furthermore, advanced treatment planning tools have been developed that allow for the simulation of magnetic fields and the visualization of the expected brain area to be stimulated. These tools assist clinicians in planning and optimizing rTMS treatment protocols, ensuring that the magnetic stimulation is delivered effectively and safely [72]. One of the most significant advancements in rTMS technology has been its integration with imaging technologies such as MRI and fMRI [73]. This integration has revolutionized the way rTMS treatments are planned and administered. By combining rTMS with real-time brain imaging, clinicians can now monitor the effects of magnetic stimulation on brain activity, allowing for adjustments to be made in real-time to optimize treatment outcomes [74]. This has been particularly beneficial in the treatment of depression, where identifying the optimal stimulation target can significantly affect the treatment’s efficacy. A study from 2020 has shown that targeting the dorsolateral prefrontal cortex (DLPFC) based on individual MRI data can enhance the treatment response in patients with major depressive disorder [75].

### 6.2. Integrating BCIs with rTMS

**Figure 5:**
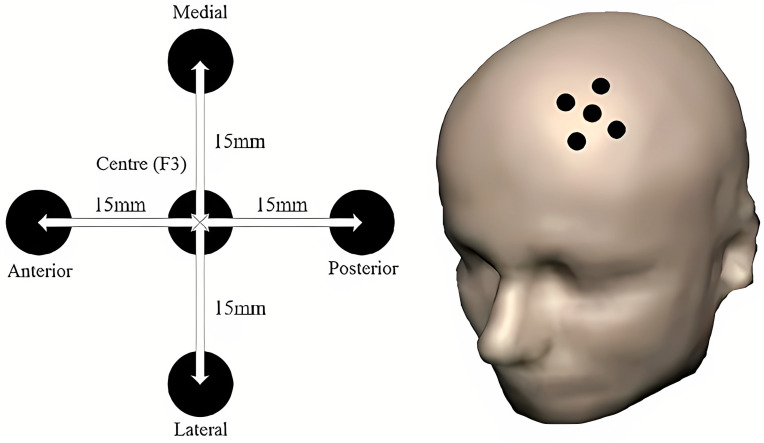
Diagram of coil positioning centered on the F3 point with 15 mm spacing between stimulation points in the medial, lateral, anterior, and posterior directions. The precise placement of TMS coils ensures targeted stimulation of specific brain regions, such as the dorsolateral prefrontal cortex (DLPFC), for more effective treatment outcomes in rTMS applications [78].

In a recent study, the potential of BCI to optimize rTMS therapy was explored [76]. While advanced treatment planning tools and the integration of rTMS with imaging technologies such as MRI and fMRI have significantly improved the planning and administration of rTMS treatments, there are limitations to these advancements [77]. One challenge is the complexity and cost associated with these technologies, which may limit their accessibility in clinics with limited resources. Additionally, the accuracy of the simulation of magnetic fields and the visualization of the targeted brain area can be affected by individual variations in anatomy and brain structure, potentially impacting the effectiveness of the treatment [77]. Integrating imaging technologies also requires specialized expertise and equipment, which may only be available in some clinical settings [78]. The reliance on real-time brain imaging during treatment also adds complexity to the procedure, requiring careful coordination and interpretation of the data to ensure optimal outcomes. Addressing these limitations is crucial for maximizing the potential of these advanced technologies in rTMS treatment planning and delivery [78]. Automation in rTMS has been significantly enhanced by AI, as seen in systems that automatically adjust treatment protocols based on neural feedback [79]. Figure 5 displays a diagram that illustrates the placement of TMS stimulation points centered around the F3 point, with a precise spacing of 15 mm in the medial, lateral, anterior, and posterior directions. This layout is often used for targeting regions such as the dorsolateral prefrontal cortex (DLPFC), an area associated with mood regulation. Optimizing the placement of these coils enhances the specificity of the stimulation, contributing to improved therapeutic outcomes in patients undergoing rTMS [79]. An example of this technology is the SmartFocus^®^ TMS technology by MagVenture (Farum, Denmark), which uses an AI-driven algorithm to adjust stimulation based on the patient’s cortical reactivity, thereby improving the accuracy and consistency of treatments [80].

### 6.3. Integration of Artificial Intelligence in rTMS Systems

Recent advancements in AI have facilitated the optimization of stimulation parameters in rTMS treatments. AI algorithms are now crucial in analyzing extensive datasets to determine ideal simulation variables tailored to individual physiological profiles [81]. This approach personalizes therapy and enhances efficacy by dynamically adjusting the pulse intensity, frequency, and coil placement. An important example is the collaboration between research institutions and tech companies to develop AI models that dynamically adjust rTMS parameters [82]. Predictive analytics, powered by machine learning, are now used to forecast patient responses to rTMS treatment. For instance, a 2021 study utilized AI to analyze data from clinical trials, successfully predicting the efficacy of rTMS in depression treatment based on patient-specific characteristics and prior response patterns [83]. However, the accuracy of AI-based predictive analytics is highly dependent on the quality of the training data, and biased data could lead to suboptimal treatment recommendations [83]. AI has also significantly improved the integration of rTMS with neuroimaging. Technologies like BrainsWay’s Deep TMS integrate AI to analyze MRI scans to optimize coil placement and stimulation areas [84]. This approach increases the precision of treatments for conditions like depression and obsessive–compulsive disorder. However, it relies heavily on the accuracy of MRI data and can face challenges surrounding integration with existing systems, leading to higher costs and potential barriers to widespread adoption [84]. AI’s ability to provide real-time monitoring and adaptive modulation of brain activity during rTMS sessions is among the most recent advancements [85]. Systems equipped with AI can now modify stimulation parameters instantly, based on the brain’s response during the session, maximizing therapeutic efficacy and minimizing side effects [86]. This development is crucial for adapting treatments to the minute-to-minute changes in brain activity. Table 3 provides a concise overview of the key innovations in rTMS software, the associated limitations, and the years they were introduced.

## 7. Case Studies and Clinical Trials

A meta-analysis from 2020 on the efficacy of rTMS in treating depression focused on preclinical studies in rodent models [87]. It systematically analyzed data to assess the effects of rTMS on depressive-like behaviors, highlighting its potential benefits. The findings suggested a significant positive impact of rTMS in alleviating depressive symptoms across different studies despite the variation in methodologies and stimulation parameters [87]. The study included 86 animals that received rTMS treatment and 74 animals that served as controls. The standardized mean difference (SMD) of 1.87 suggested a significant effect of the treatment. This underscores rTMS’s therapeutic promise for depression, emphasizing the need for further research to optimize treatment protocols and better understand its mechanisms [87].

A study from 2021 delved into the utilization of rTMS in treating bipolar disorder (BD), covering objectives, methods, results, and conclusions from a comprehensive review [88]. The primary focus was to evaluate the efficacy, safety, and tolerability of rTMS across various stages of BD, including depressive, manic, and mixed episodes, and as a maintenance treatment. This research summarized data from randomized clinical trials (RCTs), open-label studies, and case series, applying strict inclusion criteria to ensure relevance and reliability. The findings indicated mixed outcomes for rTMS in treating bipolar depression, with most studies targeting this condition [88]. Fewer studies have explored rTMS’s effects on manic or mixed episodes or its use as a maintenance strategy. Despite the promise shown in some trials, the overall evidence remains inconclusive due to limitations such as small sample sizes, study design heterogeneity, and varying rTMS parameters. Consequently, there has been a call for more rigorously designed, larger-scale studies to confirm rTMS’s efficacy in BD treatment and to comprehensively explore its potential across different BD phases [89].

Two case studies in 2021 presented details of the treatment of two patients with behavioral addictions—online pornography addiction and internet gaming disorder—using rTMS targeting the left dorsolateral prefrontal cortex (l-DLPFC) [90]. Both patients exhibited significant improvements post-treatment, with notable reductions in addictive behaviors and cravings and improvements in mood and social interactions.

- Patient 1 (compulsive sexual behavior disorder): Significant reduction in craving for porn use (from a Visual Analogue Scale score of 9 at baseline to 0 after treatment), and improvements in depression (Beck Depression Inventory-II score from 19 to 6), anxiety (Self-rating Anxiety Scale score from 50 to 35), and overall symptom severity (Global Severity Index from 75 to 41.66) [90].
- Patient 2 (internet gaming disorder): Substantial decrease in craving for gaming (from a Visual Analogue Scale score of 75 to 5), and improvements in depression (Beck Depression Inventory-II score from 13 to 4), anxiety (Self-rating Anxiety Scale score from 32.5 to 36.25), and internet gaming disorder severity (Internet Gaming Disorder Scale—Short-Form score from 32 to 0) [36]. These outcomes persisted at a 1-year follow-up, supporting the long-term efficacy of rTMS. These findings suggest that rTMS can effectively modulate neural activity related to craving, impulse control, and mood regulation, offering a promising therapeutic strategy for behavioral addictions in the absence of approved pharmacological treatments. The treatment was well-tolerated without adverse effects, highlighting rTMS as a safe and effective treatment modality for managing behavioral addictions [90]. Table 4 summarizes the findings from these recent case studies and clinical trials.

## 8. Safety, Efficacy, and Regulatory Developments

Recent updates on safety protocols and risk management in rTMS have emphasized the importance of rigorous risk analysis with any change in TMS equipment, methodology, or application context [91]. This includes updates to hardware, software, dosing algorithms, and the integration of complementary technologies like neuronavigation and imaging techniques [92]. The goal is to identify potential new risks or modify existing risk profiles, considering both adverse events and serious adverse device effects.

Improved precision in targeting and dose determination can reduce risks. However, introducing new technologies necessitates increased vigilance and adherence to established risk management frameworks, balancing potential benefits against risks [93]. Recent studies highlight the clinical efficacy of rTMS in patients with major depressive disorder (MDD) who have experienced limited treatment success with traditional medication trials. Specifically, evidence suggests that rTMS may offer significant therapeutic benefits even after ≤1 failed medication trial, challenging the conventional threshold for considering rTMS only after multiple medication failures [94]. This broader applicability could prompt revisions in coverage policies, potentially offering rTMS as a viable option earlier in the treatment process for patients with MDD [95].

Additionally, advancements in online rTMS techniques demonstrate its potential to disrupt and enhance cognitive functions across various domains, with specific parameters like frequency and targeting methods influencing the effectiveness and outcomes of the treatment [95]. Regulatory standards and approvals for rTMS have expanded significantly, with multiple agencies clearing its use for various psychiatric conditions, including treatment-resistant depression, anxiety disorders, obsessive–compulsive disorder (OCD), and smoking cessation. Furthermore, eNeura’s 2013 approval for migraine treatment highlights the expansion of rTMS applications into neurological conditions, emphasizing the versatility of this technology across therapeutic domains [95]. Recent advancements also indicate potential future applications for persistent auditory hallucinations (AH) or negative symptoms (NS) associated with schizophrenia [96]. Regulatory bodies such as the Food and Drug Administration (FDA) and CE Mark in Europe are crucial in auditing, certifying, and approving rTMS devices and protocols, ensuring their safety and efficacy based on rigorous clinical evidence [44].

## 9. Ethical Considerations

In the past five years, the use of rTMS in clinical and research settings has grown significantly. This noninvasive brain stimulation technique has shown promise in treating neuropsychiatric disorders and enhancing cognitive functions [49]. However, the expanding applications of rTMS raise important ethical considerations that must be addressed to ensure the responsible use of this technology [49].

One of the primary ethical concerns is ensuring informed consent, especially when dealing with vulnerable populations such as individuals with depression or other mental health disorders [97]. Researchers and clinicians must provide clear information about the procedure, potential risks, and benefits and ensure that consent is obtained voluntarily without any coercion. The safety of rTMS is a significant ethical concern. While rTMS is generally considered safe, it can have side effects such as headaches, scalp discomfort, or, in rare cases, seizures. It is crucial to have strict safety protocols in place and to continuously monitor and update these based on the latest research findings. There is a risk of therapeutic misuse or overuse of rTMS, particularly in the context of off-label treatments or in private clinics offering “enhancement” services [97]. Ethical guidelines must be developed to prevent such practices and ensure that rTMS is used appropriately and only when supported by scientific evidence [97].

The issue of accessibility and equity in using rTMS is also an ethical concern [98]. There may be disparities in access to this treatment based on socioeconomic status, geographic location, or healthcare coverage. Efforts should be made to ensure that rTMS is available to those who could benefit from it, regardless of their background. With the integration of rTMS with other technologies, such as neuroimaging or brain–computer interfaces, there are concerns about privacy and confidentiality [98]. It is essential to protect the personal and sensitive data of individuals undergoing rTMS treatment and to have clear policies regarding using and sharing this information [98].

While integrating AI into rTMS presents many benefits, it also introduces challenges, such as the need for high-level data security to protect patient information and prevent unauthorized access [99]. Additionally, ensuring that AI algorithms are free from bias and that their decisions are transparent and understandable remains a critical concern, especially when these decisions influence patient care protocols [100].

As the use of rTMS continues to grow, it is imperative to address these ethical considerations to ensure this technology’s responsible and equitable use [97]. Ongoing dialogue among researchers, clinicians, ethicists, and policymakers is necessary to develop comprehensive guidelines and standards prioritizing the well-being and rights of individuals undergoing rTMS treatment [97]. Table 5 summarized the recent rTMS safety developments and ethical considerations discussed in this section. 

## 10. Future Directions and Emerging Technologies

The future of repetitive transcranial magnetic stimulation (rTMS) holds significant promise, driven by rapid technological advancements and an increasing demand for effective neuromodulation therapies [103]. The global transcranial magnetic stimulation market is projected to grow substantially by 2030, reaching approximately USD 2.46 billion, driven by the rising prevalence of neurological disorders and the development of more advanced, user-friendly rTMS devices.

Future research should focus on refining rTMS device designs to address complexity, heat generation, and cost challenges. Efforts to improve the efficiency of cooling systems, optimize power circuits, and develop cost-effective materials are crucial for making rTMS more accessible and practical in various clinical settings. Modular device designs may emerge to enhance device longevity and ease of maintenance, ensuring that rTMS technology remains adaptable to evolving clinical needs [70]. Integrating artificial intelligence (AI) into rTMS systems is expected to revolutionize the personalization of treatment protocols. AI-driven algorithms will continue to optimize stimulation parameters based on individual neurophysiological profiles, improving therapeutic outcomes [104]. The market is also likely to see the rise of portable and wearable rTMS devices, which will expand the accessibility of this treatment to non-traditional settings and broader patient demographics. These developments address the growing demand for more convenient, patient-friendly neuromodulation therapies [104].

**Figure 6:**
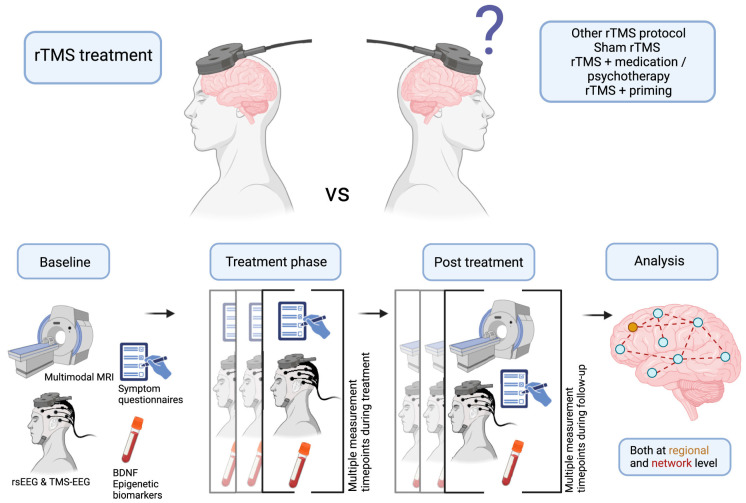
Recommendations for the design of future studies of repetitive transcranial magnetic stimulation (rTMS). Induced neuroplasticity in treating psychiatric disorders: multimodal methods, repeated measurements of multisession rTMS treatments, analysis of network-level changes, and investigations of possible methods to optimize neuroplasticity. BDNF, brain-derived neurotrophic factor; EEG, electroencephalography; MRI, magnetic resonance imaging; rsEEG, resting-state EEG [105].

Continued innovation in magnetic coil design, such as developing multi-locus and air-core coils, will enhance the precision and efficacy of rTMS treatments [105]. Future research should focus on simplifying these designs to increase their clinical applicability and reduce manufacturing challenges. Moreover, the ongoing refinement of stimulation protocols, particularly those integrating rTMS with neuroimaging and BCIs, will enable more targeted and effective treatments for a broader range of neuropsychiatric and neurological conditions [106]. Figure 6 outlines the recommended design framework for future studies investigating rTMS-induced neuroplasticity in psychiatric disorder treatments. This figure emphasizes the importance of multimodal methods, such as combining MRI, EEG, and biomarker measurements, alongside repeated assessments during multisession rTMS treatments [106]. These approaches allow researchers to analyze regional and network changes, optimizing the understanding of rTMS’s impact on neuroplasticity.

Combining rTMS with imaging technologies like MRI and fMRI has already improved treatment planning and real-time monitoring of brain activity. Future advancements in this area will likely include more sophisticated real-time neurofeedback systems, enabling clinicians to adjust treatments dynamically based on immediate brain responses. This approach could lead to more effective and safer neuromodulation therapies, particularly for complex and treatment-resistant conditions [107].

As rTMS technology evolves, addressing the ethical implications of its widespread use, particularly in vulnerable populations, will be critical. Regulatory bodies must update safety protocols and guidelines to keep pace with technological advancements, ensuring that rTMS remains a safe and effective treatment option across diverse clinical settings [108]. The projected growth of the rTMS market, particularly in regions like North America and Asia Pacific, underscores the need for ongoing innovation to meet the increasing demand for effective neuromodulation therapies. The market’s expansion will likely be driven by the adoption of new technologies, such as AI-driven systems and portable devices, which will make rTMS more accessible and appealing to both clinicians and patients [109].

While significant progress has been made in rTMS technology, several areas require further research to ensure its long-term effectiveness, safety, and accessibility. Future studies should explore the sustained outcomes of AI-driven protocols across diverse populations to ensure they remain unbiased and effective over time. Additionally, comparative research is needed to evaluate the durability of therapeutic effects between portable rTMS devices and traditional clinical systems, validating their use in remote care. Disorders such as bipolar disorder and addiction—which have shown mixed outcomes in current studies—require further investigation to establish effective maintenance protocols. Addressing algorithmic bias in AI models and finding ways to reduce the cost and complexity of advanced technologies will also be essential to expand access and equity in rTMS treatment. By closing these gaps, future advancements can lead to more reliable, accessible, and impactful neuromodulation therapies. Table 6 summarizes the future directions for rTMS discussed in this section. 

## 11. Discussion

This review highlights recent advancements in rTMS technologies, including coil designs, AI-driven protocols, and portable devices. To provide a comprehensive view of their clinical impact, it is essential to compare these innovations based on efficacy, safety, and accessibility, supported by relevant clinical trials and case studies. Clinical trials demonstrate that AI-driven rTMS protocols improve response times and remission rates for depression compared to traditional manual protocols. AI-based methods achieve a 50% remission rate versus 32% for manual approaches [88]. However, outcomes for bipolar disorder remain inconsistent, with depressive phases responding better to rTMS, while manic or mixed phases show limited improvement, suggesting the need for more tailored treatment approaches [89]. Clinical studies on rTMS have shown considerable variation in outcomes. For example, AI-driven rTMS protocols have demonstrated faster response times and higher remission rates in treating depression than traditional protocols, which require manual parameter adjustments and may be more prone to variability in results [38]. In contrast, studies on bipolar disorder have revealed inconsistent outcomes, with some phases responding better to stimulation than others [88,89]. Similarly, while AI offers enhanced precision, traditional rTMS protocols remain effective in specific cases, challenging the need for technological upgrades in all settings [89].

From a safety perspective, most innovations, such as advanced coil designs, report mild side effects, including temporary headaches and scalp discomfort [54,64]. Portable devices and improved software interfaces have minimized operational risks, making these systems safer and more user-friendly [16]. However, the challenge of algorithmic bias remains a concern with AI-driven systems. AI models rely on datasets that may not reflect the diversity of patient populations, potentially leading to biased treatment recommendations [84,85]. This issue underscores the need to continuously validate AI algorithms and integrate diverse data sources to ensure equitable care.

Accessibility is another critical consideration. While new portable devices and AI-enabled systems expand treatment options beyond clinical settings, cost and technical complexity remain significant barriers to widespread adoption [84]. Advanced neuronavigational systems improve precision but require specialized training, making them difficult to implement in resource-constrained clinics [67]. These disparities highlight the importance of balancing innovation with practicality, ensuring that rTMS remains accessible to a broad patient base.

Even with these advancements, challenges persist. Integrating AI and personalized medicine into clinical practice introduces ethical and logistical obstacles. AI systems require large-scale datasets for accurate predictions, which may not be available in all healthcare settings [64]. Additionally, the high cost of AI-enabled and portable devices limits their adoption in underfunded clinics, creating inequities in patient access [98]. Future efforts should focus on standardizing protocols and reducing the cost of advanced rTMS technologies to promote equitable access across diverse populations.

In conclusion, while rTMS technologies have advanced significantly, it is crucial to recognize the limitations and challenges associated with their clinical integration. Continued research comparing outcomes across different protocols and devices will be essential to validate these innovations. Addressing challenges related to bias, cost, and accessibility will ensure that rTMS achieves its full potential as a transformative treatment for neurological and psychiatric disorders.

## 12. Limitations

This paper has several limitations that may affect the generalizability and objectivity of its findings. The research relies on publicly available data in English, which presents a challenge as these sources may not reflect the latest proprietary innovations. Furthermore, the exclusive focus on peer-reviewed articles in English narrows the scope of the review, potentially excluding relevant research published in other languages or grey literature. Additionally, the five-year publication window (2019–2024) may have omitted older foundational studies that could provide valuable insights into the technological and clinical development of rTMS. Finally, the review was conducted by a small team of reviewers, which, despite following a structured screening process, introduces the risk of selection bias. These limitations highlight that rTMS is an evolving field, and future updated reviews are necessary. Such reviews should be more comprehensive, involve multiple reviewers, and adopt a broader inclusion strategy to reduce bias and enhance the robustness of the research.

## 13. Conclusions

Over the past five years, repetitive transcranial magnetic stimulation (rTMS) has undergone significant technological and engineering advancements, substantially improving its efficacy, safety, and application range. This review highlights these advancements, particularly in device design, treatment protocols, software integration, and the incorporation of rTMS with other diagnostic and therapeutic technologies. The evolution of rTMS has been marked by innovations such as the development of more precise and targeted coil designs, the application of AI-driven treatment protocols, and the creation of portable rTMS devices, all of which have expanded the accessibility and versatility of rTMS in clinical settings.

These advancements have enhanced rTMS’s therapeutic outcomes and addressed some limitations associated with earlier technologies, such as targeting accuracy and patient-specific treatment customization. Furthermore, integrating rTMS with neuroimaging and brain-computer interfaces represents a significant leap forward in personalizing treatments and improving their effectiveness.

As rTMS continues to evolve, future research and development will likely focus on further refining these technologies, exploring new therapeutic applications, and addressing ethical and regulatory challenges. The ongoing miniaturization and portability of rTMS devices, coupled with the potential for AI and real-time neurofeedback integration, suggest that rTMS will play an increasingly pivotal role in neuromodulation therapies, offering new hope for patients with a wide range of neurological and psychiatric conditions.

By providing a comprehensive overview of these recent advancements, this review fills a critical gap in the literature, offering a more holistic understanding of how these innovations collectively impact the field of rTMS and its clinical applications.

## Figures and Tables

**Table 1:** Innovations, limitations, and references in rTMS device design.

Innovation	Limitation	References
Improved power circuits	Increased complexity, cost, and heat generation	[45,46]
IGBT and MOSFET integration	Higher complexity, cost, and maintenance	[47,48]
Open-access TMS database	Incomplete, outdated, potential biases	[50,51]
Efficient cooling, AI for optimization, modular designs	Balancing innovation with practicality	[49,50,51]
Database enhancements	Implementation complexity, need for standardization	[50,51]

**Table 2:** Recent TMS coil advancements.

Coil Type	Year	Benefits	Limitations	References
TH Coil	2019	Deep penetration, strong magnetic field	Complex, needs further optimization	[54]
QBC	2019	Precision, reduced stimulation volume	Complex, needs further optimization	[54]
H-DC Coil	2020	Safe, efficient	No significant advantage over figure-8 coils	[55]
Air-Core Coil	2020	Improved focality, reduced eddy currents	Complex design	[56]
Circular Coil	2022	Consistent magnetic fields	Needs more comparative data	[57]
Multi-Locus Coil	2023	Faster optimization, precise control	Manufacturing challenges	[59]

**Table 3:** Summary of innovations in rTMS software from 2019–2024, control systems, and their limitations.

Innovation	Limitation	References
Neuronavigation with MRI for precise coil positioning	Complex, needs training	[66,67]
Advanced software for precise stimulation control	Standardization, reliability issues	[68,69]
User-friendly touch-screen interfaces	Cybersecurity risks	[70,71]
Tools for simulating and visualizing target areas	High cost, compatibility issues	[68,72]
Real-time adjustments using MRI/fMRI	Complex, specialized equipment needed	[73,78]
AI-driven real-time treatment adjustments	Increased complexity	[79,86]
AI-based predictive analytics for personalized treatment	Data quality, bias risk	[81,83]
AI improves precision via MRI scan analysis	MRI accuracy, integration, cost	[84,85]

**Table 4:** Recent rTMS Studies on depression, bipolar disorder, and addictions.

Study Focus	Findings	Limitations	Refs.
2020 Meta-Analysis on Depression	rTMS reduces depressive behaviors	Method variation	[87]
2021 Study on Bipolar Disorder	Mixed results for depression	Small samples	[88,89]
2021 Case Studies on Addictions	Reduced cravings, improved mood	Very small sample	[90]

**Table 5:** Recent rTMS safety developments and ethical considerations.

Topic	Key Points	Refs.
Safety Protocols	Rigorous risk analysis for rTMS changes	[91,92]
Efficacy in MDD	Effective after ≤1 failed medication trial	[95]
Online rTMS	Enhances cognitive function; frequency matters	[95]
Regulatory Approvals	Expanded psychiatric use; future schizophrenia use	[96,101]
Informed Consent	Clear, voluntary consent, especially for vulnerable	[49,97]
Side Effects	Monitor to prevent headaches, seizures	[97,102]
Misuse	Prevent off-label and enhancement misuse	[97,98]
Accessibility	Address access disparities	[97,98]
Privacy	Protect data with tech integration	[97,98]
AI Security	Ensure secure, unbiased AI	[99,100]

**Table 6:** Future directions for rTMS.

Future Directions in rTMS	Key Points	Refs.
Market Growth	rTMS market to hit USD 2.46 billion by 2030	[103,110]
Device Design	Simplify, cool, and cut costs; modular designs	[70,104]
AI Integration	AI for personalized, portable treatments	[70,104]
Coil Technology	New coil designs; ties with neuroimaging, BCIs	[105,106]
Imaging & Neurofeedback	MRI/fMRI with real-time neurofeedback	[107,108]
